# Invisible (bio)economies: a framework to assess the ‘blind spots’ of dominant bioeconomy models

**DOI:** 10.1007/s11625-023-01292-6

**Published:** 2023-02-01

**Authors:** Lilian Pungas

**Affiliations:** 1 Institute for Sociology/Junior Research Group Flumen, Friedrich Schiller University, Jena, Jena, Germany; 2 Berlin, Germany

**Keywords:** Bielefeld subsistence approach, Food Self-Provisioning, Capitalist nature relations, Socio-ecological transformation, Decolonial ecofeminisms, Iceberg model

## Abstract

Bioeconomy as a new promissory discourse neither challenges the paradigm of economic growth, nor questions its embeddedness in capitalist (neo-)colonial patriarchal power relations. However, the calls for a ‘genuine’ socio-ecological transformation and for alternative bioeconomy visions imply exactly a destabilization of these power relations. Drawing on the Bielefeld subsistence approach and on its colonialism–capitalism–patriarchy nexus, I argue that the latest bioeconomy strategy and policy papers of both the EU and Estonia each disregard certain spheres of the bioeconomy due to the three-dimensional power relations. As a seemingly neutral political discourse, the bioeconomy is shaped by cultural assumptions and narratives that determine and perpetuate what is deemed worthy of protection and what is pushed aside as merely ‘natural’. As such, the current bioeconomy papers promote a ‘biomass-based model of capital accumulation’ that is essentially built on the prerequisite of the subordination, devaluation, appropriation and/or exploitation of (1) different geographical regions, (2) ecological foundations, and (3) prevalent bioeconomy practices. As a widespread agricultural practice in Eastern Europe, Food Self-Provisioning (FSP) serves as a good example of how predominant bioeconomy models (1) simply operate as new forms of postcolonial development discourse, instead of embracing the plurality of decolonial ‘alternatives to development’; (2) deepen the human–nature dichotomy by regarding nature as a mere resource to be extracted more efficiently instead of cultivating mutually nourishing partnership-like relation(ship)s with nature; and (3) maintain the separation between monetized and maintenance economies, rather than fostering ethics of care to overcome the structural separation between the latter.

## Introduction

With increasing global challenges, such as anthropogenic climate change and exacerbating environmental damage, the emerging bioeconomy—an economy primarily based on renewable biological resources—seems to provide a bright vision of green(er) economic growth, a welfare state with high social standards as well as environmental protection (EU 2018, p. 8). Accordingly, it is only natural that governments and international organizations (EU 2012, 2018; OECD 2018) have welcomed bioeconomy with its attractive ‘triple-win’ promise as a ‘panacea for all ills’ (“European way: being economically viable with sustainability and circularity in the driver’s seat” EU 2018, p. 7) and its aim to secure (or increase) economic competitiveness in this ‘new’ economic sector based on biogenic resources. However, the founding figure of Ecological Economics, Georgescu-Roegen, who initially termed the concept ‘bio-economics’, pointed out to the ecological foundation of all economic activities and consequently to the inevitability of physical and material boundaries. Various environmental initiatives, think tanks, civil society organizations (e.g., Mills 2015; Civil Society Action-Forum on Bioeconomy/denkhausbremen 2019; Biofuture Platform 2016) and scholars (Birch et al. 2010; Birch and Tyfield 2012; Backhouse et al. 2021; see also Eversberg et al. 2022) have thus criticized the promissory discourse on bioeconomy as a mere ‘greening’ of the current economic model by “hijacking” the term bioeconomy (Vivien et al. 2019).

**Fig. 1:**
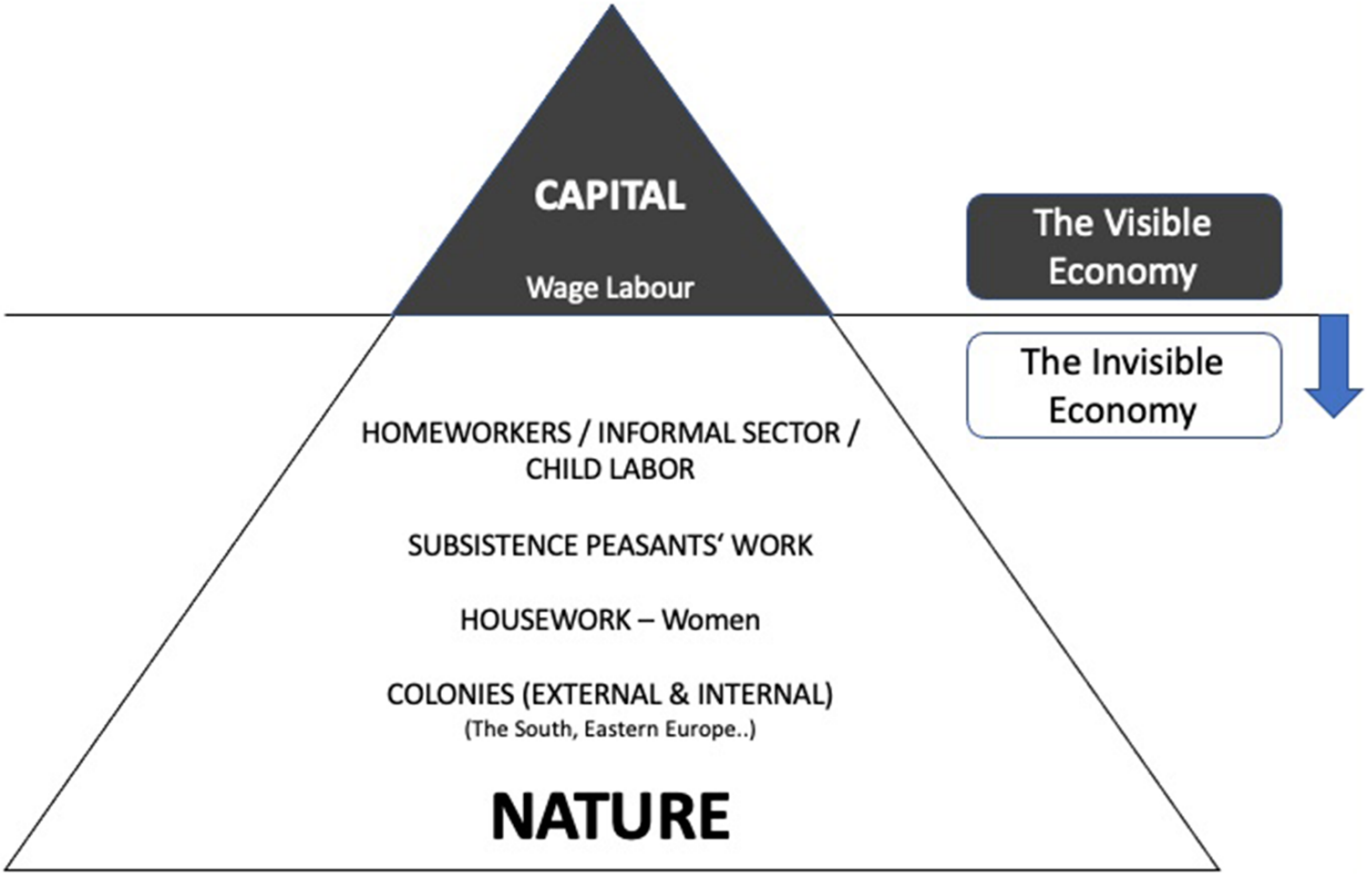
Maria Mies’s iceberg model of capitalist patriarchal economies, demonstrating the ‘structure of separation’ as well as the ‘tip’ and ‘bottom’ parts of the iceberg (own depiction, adapted from Mies 2007, p. 271). Similar ideas have been visualized in Hazel Henderson’s Layer Cake with Icing (1982), in which the monetized market economy is merely the upper layer, the so-called Icing

In this article, I will analyze the Food Self-Provisioning (FSP) practice against the backdrop of the bioeconomy strategy and policy papers of the EU and Estonia. FSP as a practice of “growing and consuming one's own food using one's own (predominantly non-monetary) resources” (De Hoop and Jehlička 2017, p. 811) takes place outside the conventional agri-food system. Despite being an established practice in Central and Eastern Europe (CEE) and as such also serving as a prevalent agricultural sector of the bioeconomy, it remains ‘invisible’ in bioeconomy papers. My study interest is, therefore, to explore whether the Bielefeld subsistence approach by Mies et al. (1988) could be applied to this case and if so, whether it can enhance our understanding of the reasons for the invisibility of certain bioeconomy practices, such as FSP in CEE. The three-dimensional colonialism–capitalism–patriarchy nexus of their approach highlights how the visible part of the economy—feminists have called it the tip of the iceberg^1^ (Fig. 1)—and hence capitalism, along with capitalist primitive accumulation, crucially depends on colonial and patriarchal appropriation of what lies beneath the surface: women, nature and colonies. Seen through the theoretical lens of the Bielefeld subsistence approach, which explains global interrelations and interdependencies, these bioeconomy papers, therefore, appear to focus merely on a small part of the (bio)economy, leaving out all other parts (which I conceive as ‘blind spots’). As such, the main research question is as follows:

**Can the Bielefeld subsistence approach explain why certain parts of the bioeconomy remain ‘blind spots’ (such as FSP) in current Estonian and European bioeconomy policy papers, and if so, how?**

To my best knowledge, and despite the huge variety of criticism on the EU bioeconomy strategy (2018), there is no analysis to date that addresses the prevalent bioeconomy practices that are excluded from the bioeconomy papers. However, feminist critique on Green Economy or predominant bioeconomy models has been voiced by scholars, such as Salleh (2010, p. 209, 2020), Lettow (2013), Sinaga (2021) and Saave et al. (2022), among others. I base this article on an abductive analysis (Danermark et al. 2005) in which I first identify and explore the phenomenon under observation (the invisibility of FSP in bioeconomy policy papers). I then elaborate on the context in which FSP takes place as well as on what bioeconomy papers have to say in regard to agriculture. In a second step, I will relate this phenomenon to the Bielefeld subsistence approach. This recontextualization allows me to develop a framework that explains the invisibility of FSP in EU and Estonian bioeconomy papers from a new angle. I will apply this three-dimensional framework to the case of FSP invisibility in more detail in the respective sections, before concluding with a short discussion.

## FSP in the context of Estonian and EU bioeconomy papers

In this section, I will draw on the example of FSP in Estonia and demonstrate how certain bioeconomy practices such as FSP exist neither in the wide selection of the Estonian bioeconomy strategy and policy papers nor in the EU bioeconomy strategy (2018).

Despite various studies and policy papers on Estonia’s bioeconomy, the overarching Estonian bioeconomy strategy paper has not yet been published. National bioeconomy sectors are expected to be guided mainly by the sectoral strategy papers (e.g., forestry, agriculture, and fishing). “The Estonian Agriculture and Fisheries Development Plan 2030”—referred to here for simplicity as the Estonian agri-bioeconomy paper (Agri 2021)—currently functions as the agricultural bioeconomy strategy in Estonia. Below is an overview of all the Estonian and EU bioeconomy policy and strategy papers analyzed with special regard to their diagnosis of current problems and future goals in the agricultural sector. Notably, none of the documents mentions practices, such as FSP (or home-gardening, self-produced or self-grown food, or else) (Table 1).

**Table 1:** Overview of the analyzed bioeconomy policy and strategy papers of Estonia and EU, including their main foci, especially with regard to the diagnosis and goals in the agricultural sector

EE or EU	Document	Authors/project	Year	Foci, suggestions and future goals/strategies with regard to the agricultural sector of the bioeconomy
EE	Memorandum for the Cabinet Meeting. (Memorandum valitsuskabineti nõupidamisele)	Peepson, Argo	2022a	Maintaining and increasing economic competitiveness (p. 2) Increasing social welfare (p. 2) Innovation and R&D (p. 2) Foreign investments (p. 2) More added-value sectors (e.g., biotechnology, digitalized agriculture) (p. 2)
EE	Analysis and proposals for the development of bioeconomy. Document for the Government (Analüüs ja ettepanekud biomajanduse arendamiseks)	Peepson, Argo	2022b	Maintaining and increasing economic competitiveness (p. 2, 12) Innovation and R&D (p. 2, 12f) More added-value export (p. 2, 8f), especially with regard to agricultural products (p. 10, 18) More added-value sectors (e.g., biotechnology, digitalized agriculture) (p. 2)
EE	ADDVAL-BIOEC Final Report. Adding value and using raw materials more efficiently in the Estonian bioeconomy. (Lisandväärtuse tõstmine ja toorme tõhusam kasutamine Eesti biomajanduses. ADDVAL-BIOEC uuringu lõppraport)	ADDVAL-BIOEC	2021	More added value and efficiency (e.g., the title itself, p. 69) Better use of raw material (p. 55) Increasingly complex economic activities (p. 44, 69), incl. IT, technological innovation Biotechnology (p. 45, 54) More international cooperation in the Baltic Sea Region (p. 46, 69)
EE	“What will Estonia's bioeconomy look like in 2050?” (Summary of the scenario and foresight work for the project ‘ADDVAL-BIOEC: adding value and making more efficient use of raw materials in the bioeconomy and its sectors’)	Tiits and Karo	2021	Four future development paths of global/Estonian bioeconomy Increase of labor productivity and complex products for export (p. 2, 9f) Co-designing international agreements and standards (p. 2, 10) Scientific and technological innovation (p. 2ff, 6) Biorevolution and presence at the forefront (p. 10)
EE	Estonian bioeconomy development scenarios 2030–2050 (Eesti biomajanduse arengustsenaariumid 2030–2050), Research project ADDVAL-BIOEC, Report of the 2.3 and 2.4 Work Packages	Tiits et al.	2021	Increase of labor productivity and complex products for export (p. 5) Technological innovation and R&D (p. 69) Biotechnology (p. 42, 46, 63, 68) Diagnosis: too much export of raw biomass in agriculture (p. 15f) Organic agriculture: rising trend due to customer preference (p. 66, 99)
EE	The Estonian Agriculture and Fisheries Development Plan 2030 (Põllumajanduse ja kalanduse valdkonna arengukava aastani 2030)	Agri (Maaeluministeerium)	2021	Preference for Estonian food, less imports (p. 7) Environmental protection and biodiversity (p. 5) Competitive producers, increased exports (p. 7) Diagnosis: too much export of raw biomass in agriculture (p. 6) Cascading, reducing food waste or adding value to by-products (p. 7, 44ff) Low productivity in organic farming despite high share of land (p. 13) Organic agriculture: rising trend due to customer preference and increased awareness (p. 6)
EE	Analysis of the Estonian bioeconomy (Eesti biomajanduse analüüs)	Arengufond	2015	More added-value production (and export), increased labor productivity (p. 4, 30) “Bioeconomy’s potential is big” (p. 4) Reducing food waste or adding value to by-products (p. 16, 32)
EU	A sustainable Bioeconomy for Europe. Strengthening the connection between economy, society and the environment: updated Bioeconomy Strategy	European Union/Directorate-General for Research and Innovation	2018	Achieving sustainability by modernizing industry (p. 4) Strengthening European competitiveness with innovation (p. 4, 22, 27, 53) Improving and innovating the food production and consumption (p. 4), e.g., cutting food waste by 50% (p. 6) News jobs and employment (p. 4ff, 10) Biotechnology (p. 5, 42) Ensuring food and nutrition security as the first objective (p. 8, 22, 26) Supporting and promoting all types of innovations and (best) practices for sustainable food and farming systems (p. 12, 46ff, 75), incl. Research and Innovation investments (p. 48)

The implementation of bioeconomy in the agricultural sector in both the EU and Estonia is expected to lead primarily to the increase of labor productivity and added-value production (incl. food waste and by-products), technological innovation and cascading. There appears no critical questioning of structural aspects of the current agri-food systems, such as problematic meat and dairy farming practices, consumption or fertilizing levels (Ekardt et al. 2021; Levidow et al. 2012; Värnik et al. 2021). However, as the global agri-food system currently accounts for about 30% of greenhouse gases (not to mention its other grave impacts on the environment) (Clark et al. 2020), it has to undergo major transformation for the sake of ecological sustainability. Despite widespread criticism of the European Common Agricultural Policy (CAP) for failing to take into account socio-ecological challenges (Heyl et al. 2020; Scown et al. 2020), there is concern that current bioeconomy policies will exacerbate them even more. It is feared that by increasing the pressure on the land (e.g., with additional land use for conventional bioenergy production), socio-economic inequalities within and outside of Europe will intensify (Hennig 2017, p. 12, Backhouse et al. 2021).

Despite the demands for systemic change, the solutions proposed remain limited to only one model. In a nutshell, this can be termed a biomass-based model of ecological modernization with a highly problematic belief in “technofixes” and decoupling, keeping alive the promise of further growth and expansion (Eversberg et al. 2022; Levidow et al. 2019; Bugge et al. 2016; Birch et al. 2010; Parrique et al. 2019; Wieding et al. 2020). As Goven and Pavone (2015) argue, this model of bioeconomy should be understood both as a political project (and not simply a technoscientific one) and as a response to the acute challenges inherent to the current neoliberal-capitalist accumulation regime, that aims to protect and extend itself using bioeconomy simply as a means to an end. As a “master narrative”, the bioeconomy fuses technological advance with societal progress (Delvenne and Hendrickx 2013, p. 75).

In the case of Estonia, the agricultural bioeconomy sector accounted for around 3% of employment and GDP in 2019 (Agri 2021, p. 43). However, according to the Agricultural Census 2011 (Valdvee and Klaus 2013), every third household has been involved with FSP practices, with a slightly decreasing tendency according to the latest census (Stat 2021). As organic agriculture made up 20% of the arable land (ibid, p. 36) but produced about 8% of the agricultural value (Agri 2021, p 43), the agri-bioeconomy paper emphasizes the potential of organic agri-food markets in Estonia and compares the country’s organic consumption (1% in 2018) with that of the “World’s leading organic nation” Denmark (13.3% in 2017) (ibid, p. 44). Not mentioned is the wide-spread organic food production or consumption that takes place *outside* the market.

However, looking at the practice of FSP, a completely different picture emerges. Here, Estonia could probably be considered—at least in Europe—as one of the ‘leading organic nations’ among other post-socialist countries. As various studies demonstrate, not only is FSP a vivid socio-cultural practice in CEE countries (Vávra et al. 2018), but the amount of self-grown and consumed food outweighs that of Western countries significantly. In 2003, 59% of the population in Slovakia and 41% in Estonia engaged in the so-called ‘informal food production’ (that I equate here with FSP), in contrast to 6% in Denmark and 5% in the Netherlands (Alber et al. 2003, 11f). Various scholars have demonstrated multidimensional motives for, and benefits of, this practice in Poland (Smith et al. 2015), Hungary (Balázs 2016), Czechia (Sovová et al. 2021), Croatia (Ančić et al. 2019), Baltic countries (Mincytė 2011; Aistara 2015; Pungas 2019) and Moldova (Piras 2020). These agricultural practices (often including crop rotations with intercrops, such as legumes, organic fertilization, composting and green manure), have a positive impact on soil health and biodiversity, thus serving as an example of “quiet sustainability” (Smith and Jehlička 2013) and “quiet food sovereignty” (Visser et al. 2015).^2^

As such, FSP can be regarded as a case and curious phenomenon of the invisible bioeconomy in the agricultural sector. Despite its prevalence and socio-ecological benefits, it does not seem to be worth mentioning in the bioeconomy policy papers. In the following sections, I will explore why this is the case.

## The Bielefeld subsistence approach and framework to assess the ‘blind spots’ of the dominant bioeconomy models.

My suggested theoretical framework is built on the work of Claudia von Werlof, Maria Mies and Veronika Bennholdt-Thomsen (1988), known as the Bielefeld subsistence approach (see also Bennholdt-Thomsen and Mies 2000). The authors developed the Bielefeld subsistence approach in their collection of essays called “Women: the last colony” (1988). In this publication, they examine the systematic relationship between the “unseen foundations” (ibid. 1) of the global capitalist-patriarchal model of accumulation (women, colonies, and nature) and their ongoing exploitation and appropriation. In doing so, they draw on the work of Rosa Luxemburg (Luxemburg 1913, 2015), world-systems theory (Wallerstein 1974, 1979), feminist domestic labor debates (Federici 1975, 2004) and the environmental movement.

The subsistence approach diagnoses a systematic exclusion of three spheres that in capitalism are regarded as ‘Nature’—including women (or mostly female housework), land (or natural resources) and the colonies—and as such are “free for unrestricted appropriation” (Mies et al. 1988, p. 8). Despite constituting the invisible foundation of all production processes, the exclusion of these spheres through the “structure of separation” (Biesecker and Hofmeister 2010) is a necessary precondition for the present-day operation of capitalism. This “structure of separation” draws an artificial line between those parts of the economy that are considered ‘productive’ parts of the (bio)economy. Other parts are neglected or made invisible—to legitimize their devaluation and appropriation (Saave 2022). The consequent and in most cases violent appropriation is legitimized with the antagonistic framing of nature (including the ‘naturalized’ objects—women and colonies) as a ‘backward’, ‘savage’ and ‘primitive’ object, standing in sharp contrast to the ‘civilized’, ‘progressive’ Western counterpart as a subject (Plumwood 1993; Federici 1975, 2004). The authors extend Rosa Luxemburg’s (1913, 2015) analysis on capital accumulation by arguing that women, colonies, and nature are not merely the so-called “non-capitalist strata and milieux” (Mies et al. 1988, p. 6). Instead, they are both a main target *of* and a precondition *for* the process of ongoing primitive accumulation. This again is based on the exploitation of wage labor and results in diverging and exploitative class relations within all societies.

Although the Bielefeld subsistence approach is novel in determining the three ‘colonies’, the theory of triple oppression or triple exploitation (class, race, gender) by Claudia Jones and Louise Thompson Patterson problematized similar aspects already in the 1930s (Lynn 2014). The books by Angela Davis (Women, Race and Class, 1981) as well as bell hooks (Ain’t I a Woman? Black Women and Feminism, 1981) laid the basis for the so-called Integrative RGC (Race Gender Class) Studies. Intersectionality as an analytical framework has been developed based on equal recognition of the neglect and subordination of persons due to their interlocking ‘disadvantageous’ characteristics (in addition to class, race, gender also sexuality, religion, caste, disability). In the same way as the subsistence approach, intersectionality suggests that different ‘axes of oppression’ cannot be examined (and overcome) in isolation from each other, because they mutually reinforce each other. Marxist ecofeminists thus argue for common political goals of feminist, socialist, ecological, and indigenous struggles against transnational capital (Salleh 1997).

The Bielefeld subsistence approach has been criticized for its essentialism (Agarwal 1992; Braidotti et al. 1994), ethnocentrism (Mohanty 1988), and for introducing a further antagonism between household work/subsistence and wage labor. Instead of overcoming the prevalent dichotomies and expanding the analysis by integrating the ‘invisible foundations’, the approach devalues and downgrades both wage labor and men, thus reproducing the same structural division it accuses its counterparts of. Despite this valid criticism, the Bielefeld approach still provides an analytically valuable basis for examining systemic and mutually reinforcing power relations as well as structures of devaluation, oppression, and appropriation.

**Fig. 2:**
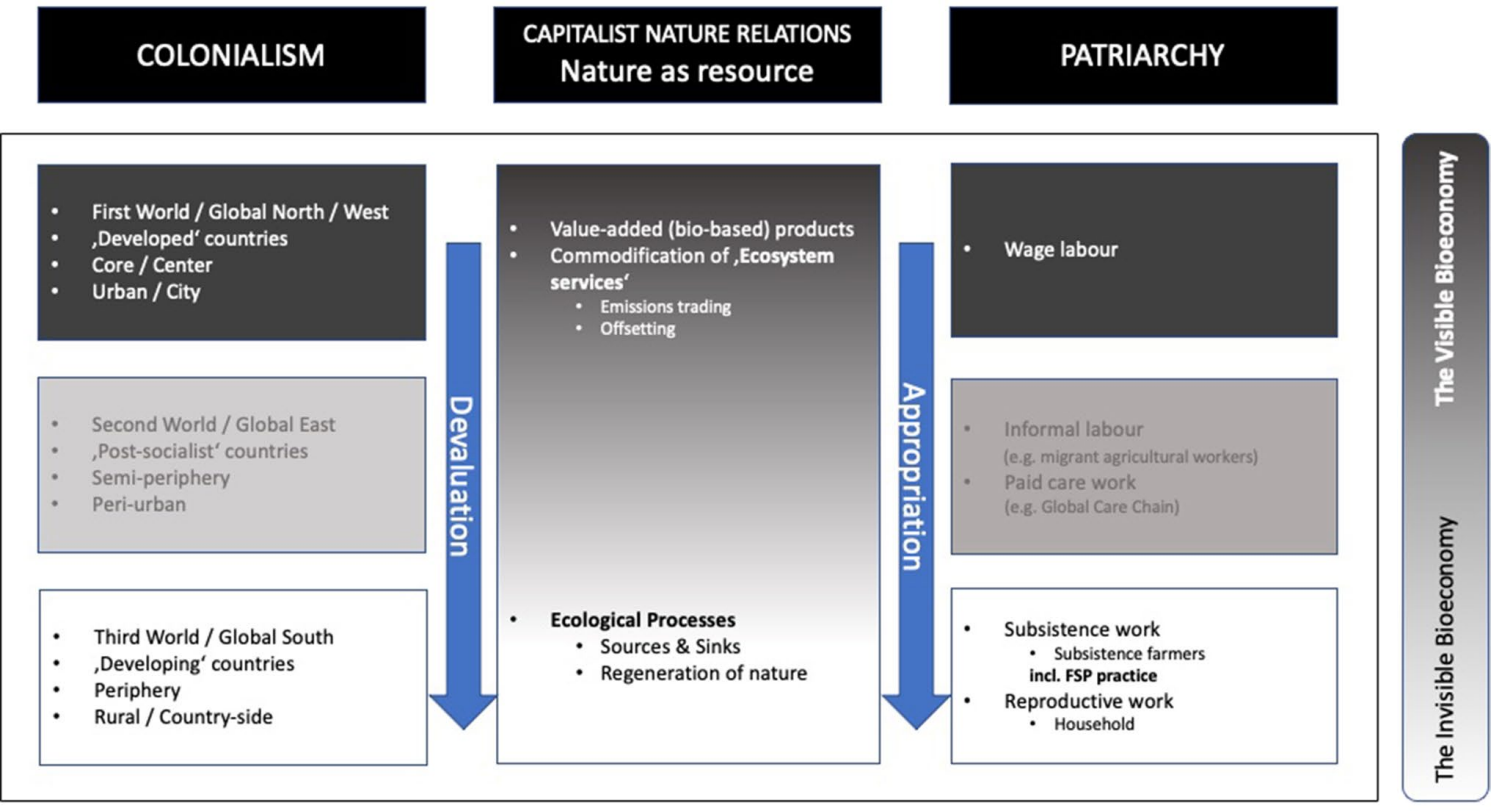
Framework to assess the ‘blind spots’ of the dominant bioeconomy models. Adapted from the Bielefeld Subsistence approach and Coloniality–Capitalism–Patriarchy Nexus (Mies et al. 1988)

Building on the empirical data from bioeconomy policy documents and interviews with city and Ministry officials, I have applied the Bielefeld subsistence approach to the phenomenon of ‘FSP as an invisible bioeconomy practice’. As part of an abductive step of synthesis, I moved back and forth between the empirical data and the applied theories in an iterative process (Danermark et al. 2005:, p. 112, Tavory and Timmermans 2014). The result of recontextualizing this specific phenomenon using the Bielefeld subsistence approach can be seen below as a framework that highlights the three-dimensional power relations constituting the ‘invisibility’ of FSP as a bioeconomy practice (Fig. 2).

In the framework, three spheres (colonialism, capitalist nature relations and patriarchy) each demonstrate either a clear or diffuse hierarchy between (1) what is considered as valuable, globally desirable or is seen as part of the ‘formal’ and ‘visible’ economy and (2) what is perceived as outdated, inefficient, labelled as ‘informal’, subsistence or ‘taken for granted’ as an unrestricted resource meant for appropriation. Between the visible and invisible parts there are the liminal ‘grey’ spheres (see also Müller 2020). With regard to ‘FSP as an invisible bioeconomy practice’, this framework suggests the following theses, all of which will be elaborated on in detail in the subsequent sections:

- In the colonialism sphere, the promoted bioeconomy models essentially serve the needs of the Western economies of the Global North. The knowledge about bioeconomy and its practices that originate from this region are promoted, while others are devalued. Furthermore, the material flow demonstrates a tendency for deepening agrarian extractivism (3).
- In the sphere of ‘capitalist nature relations’,^3^ nature is treated as an endless reservoir of resources, and a provider of ecosystem services for human needs. Despite all promises of ‘greening the economy’, this anthropocentric view prioritizes profitable technoscientific solutions, while more radical and eco-centric answers addressing the root causes of current challenges are marginalized (4).
- In the patriarchy sphere, bioeconomy models focus exclusively on formal wage labor, as opposed to unrecognised housework and subsistence farming, which both constitute an essential part of the ‘invisible’ bioeconomy (5).

In the following sections, each sphere will be elaborated on with regard to the specific forms and characteristics of its devaluation and appropriation. I will bring together theoretical positions of the Bielefeld subsistence approach with the case of FSP to understand the reasons for its invisibility in bioeconomy policy papers.

### Colonialism—bioeconomy as just another development discourse?

The first sphere “Colonialism” encompasses the critique of the world-systems theory by Immanuel Wallerstein (1974, 1979), as well as post-development, post- and decolonial discourses. As demonstrated by various scholars, bioeconomy in its current implementation is often accompanied by serious environmental harm (deforestation, biodiversity loss), and an increase of socio-economic injustice and violence (land use and access conflicts due to green grabbing, eviction of local communities from their land, and destruction of their livelihoods) in the global peripheries (Backhouse et al. 2021). As such, the current EU bioeconomy strategy arguably perpetuates (or even exacerbates) green colonialism in a comparable way as, for instance, the European Green Deal or American Green New Deal have been accused of doing (Zografos and Robbins 2020; Fuchs et al. 2020; Basu 2021; Eberle et al. 2019; Lyons and Westoby 2014). Therefore, it can be regarded as a neocolonial strategy to maintain the EU’s global leader status and decarbonize its economy, while simultaneously devaluing the knowledge and traditions originating from the (semi-)peripheries, appropriating their resources and/or undermining their livelihoods.

According to the world-systems theory, the world system is divided into (mutually dependent) core, semi-peripheral, and peripheral countries with distinct characteristics (high skill, capital-intensive manufacturing vs. low skill, labor-intensive production accompanied by the extraction of raw materials). This order reinforces the dominant position of the core countries over other countries. It is argued that there cannot be ‘one without the other’—core countries depend on the raw materials of the periphery (and thus on the fact that there always will be a periphery to extract from), whereas the periphery depends on the core’s capital. Semi-periphery, in this case, is positioned between the core and the periphery, exhibiting features of both, and operating as an intermediary (Chase-Dunn and Hall 1997; Flint and Taylor 2018). As a liminal space in-between, post-socialist Europe (the previous ‘Second World’ or “Global East”) (Müller 2020), for instance, is considered a semi-periphery (Domazet and Jerolimov 2014). This positionality can also be found in the urban–rural divide, where centers tend to dominate rural areas with regard to policies and future trajectories, yet in many cases depend on the resources of the latter (Moore 2003, p. 452, “Cities should become major circular bioeconomy hubs” EU 2018, p. 6).

For legitimizing and perpetuating the neo-colonial dominance and exploitation, the core countries (or centres) command a specific ‘developmentalist’ vocabulary. In other words, the only proclaimed way ‘out’ of the (semi)peripheral status is ‘development’ which will be “modelled on the European precedent” (Mies et al. 1988, p. 3) and designed, measured and monitored by their indicators and institutions (Jehlička and Jacobsson 2021). Walter Mignolo has, therefore, conceptualized the relationship between modernity and coloniality as constitutive of each other (Mignolo 2000, 2011). Exploring Soviet coloniality as co-constituted with Soviet modernity, Epp Annus (2017) argues that a postcolonial analysis should focus on the specific ways in which a “colonial situation conditions the sphere of the possible within a given society, how it guides its subjects’ aims and desires in certain directions and closes off others, how it encourages certain interpretative models over the others [..]” (ibid, p. 88). Ultimately, the cultural, political, and economic legacies of colonialism tend to get perpetuated through the “Catching-up narrative” or promises of ‘development’.

Although the framing of ‘development’ or modernization^4^ as a ‘panacea for all ills’, as universally desirable, or as a means to an end (for welfare, overcoming poverty and hunger) has been criticized by various post-development scholars for the concept’s euro-centric and authoritarian implications (Ziai 2016, p. 54; Ziai 2015; Escobar 2015), the Global North (the EU in this example) operates (once again) as an ‘active’ agent, determining paths, conditions, and promises for the envisioned transition to the bioeconomy (Vogelpohl and Töller 2021, p. 144; EU 2018, p. 46). The ‘passive’^5^ peripheral countries in the Global South and semi-periphery are expected to adhere to this model and apply the same logic when designing their own bioeconomy strategies. However, in this way, the very same political–economic structures, technocratic actor coalitions (between banking, biotech, agribusinesses, and energy corporations) and techno-scientific solutions are perpetuated. Second, this (self) proclaimed “exclusive knowledge” (Inayatullah 2014)—meaning, for instance, the Global North deciding which knowledge counts as the knowledge in knowledge-based bioeconomies (Birch et al. 2010; Backhouse et al. 2021, 29ff)—tends to reproduce governmentalities aimed at “emulating, imitating, “cloning” or conforming to hegemonic models” (Figueroa Helland and Lindgren 2016, p. 433). The consequent discursive maltreatment (Kuus 2004) of other forms of knowledge contributes to epistemic injustice and violence, and thus not only hinders the plurality of knowledges originating from the (semi-) peripheries (Müller 2020; Jehlička 2021; Delvenne and Kreimer 2017), but is by itself a neocolonial practice (Demeter 2019, Schott 1998).

Furthermore, for the EU and Estonia alike, the proclaimed goal of the bioeconomy strategy is to secure (or increase) economic competitiveness, ensure exports and maintain global leadership in global bioeconomic markets and governance (EU 2018, p. 6, 10, 47; Peepson 2022a, b, p. 2; Agri 2021, p. 7). By contrast, the peripheries seem to be perceived as unlimited reservoirs of biomass to satisfy the needs, desires (and the greed) of the import-reliant core countries or cities (Paul 2013, p. 18; Hall and Zacune 2012, p. 4; Levidow 2013)—now in a ‘green’, carbon–neutral way, e.g., when wood pellets from Eastern Europe are burnt in power plants in the Netherlands to decarbonize the Dutch economy (Fuchs et al. 2020; van der Wal 2021).

### Colonialism: “FSP is simply an outdated ‘survival strategy of the poor’”

By re-contextualizing the specific phenomenon of interest—the invisibility of certain bioeconomy practices, such as FSP in CEE—within this framework, I am able to reveal the continued stigmatization, devaluation, and appropriation of this practice along the lines of postcolonial logic. Unlike the EU and Estonian bioeconomy papers, with their proclaimed goals of economic growth and techno-scientific advances, FSP neither provides ‘novel value-added products’ nor promises Estonia to “reach the top of the world’s most prosperous countries” as the nationally promoted bioeconomy model does (ETA 2021).

Instead, it is often regarded as yet another example that proves the urgent necessity of Western ‘modernization’, thus legitimizing the postcolonial narrative of ‘catching-up’. Within the agricultural sector, Estonia’s main focus, therefore, lies on leaving behind the status of a semi-peripheral raw biomass exporting country and ‘struggling up’ to reach a core country status (just as, e.g., Scandinavian countries to which comparisons are repeatedly made, see ADDVAL-BIOEC 2021, 22ff; Peepson 2022b, p. 4, 9). Bioeconomy is simply one means by which Eastern European countries should aim to achieve this goal (EU 2018, p. 31). As such, ever more export of increased value-added products is what supports the main agenda in Estonia (Rohegeenius 2021; Peepson 2022b, p. 2, 8f; Tiits and Karo 2021, p. 2, 9f) rather than diverse small-scale, community-based and sufficiency-oriented agricultural practices.

What devalues FSP as an agricultural practice in Estonia even more is its association with the past Soviet era. This leads to the common interpretation of FSP as a ‘survival strategy of the poor’, as shown by article titles, such as “Muddling through economic transition with garden plots” (Seeth et al. 1998; see also Alber and Kohler 2008; Ries 2009). These associations explain the ongoing postcolonial devaluation of the practices and knowledges originating from the ‘Second’ or ‘Third’ World (Jehlička and Jacobsson 2021). The ‘First World’ appears to have gained the superior knowledge, while Eastern knowledge is thought to be “irrational, unscientific, traditional and subjective” (Inden 1986, p. 408; Inayatullah 2014; Herrschel 2001). However, FSP as a form of (mostly) organic farming is a highly knowledge-based form of agriculture, involving traditional remedies, inherited and acquired skills and know–how (Vávra et al. 2018; Pungas 2019; Niggli et al. 2008, p. 34). Western scholarship, the bioeconomy policy papers, and even activists commonly oversee this valuable FSP knowledge base—a fact that stands in sharp contrast to rather popular ‘Western’ labels, such as permaculture or agroecology, which are indeed at least shortly mentioned in respective papers (EU 2018, p. 48; Agri 2021, p. 56; Peepson 2022b, p. 17). It seems that Western “niche practices” in the agricultural sector outweigh their wide-spread Eastern counterparts (such as FSP). The promise of bioeconomy to “develop the knowledge-base for a sustainable increase in primary production, taking into account all options from cutting-edge science to local and tacit knowledge” (EU 2018, p. 9) then falls short, since the “local and tacit knowledge” originating from CEE countries does not seem to be relevant enough to be mentioned in any of the bioeconomy papers with regard to food security, food sovereignty, or healthy and environmentally friendly food production.

It is equally important to state that the postcolonial narratives and images of valuable vs. outdated bioeconomy or agricultural practices get perpetuated also within Estonia as “colonial matrices of power” (Annus 2017, p. 87). Unlike fashionable community garden projects (a rather ‘Western’ idea of urban agriculture), which are often located in the city centres, advertised for the sake of Green Capital awards and financed by the municipality, FSP takes place in the (semi-) peripheral areas of the country. FSP gardeners experience a threefold ‘peripheralization’ as they are located on the downside of the respective urban–rural, center-peripheral, and east–west divides (Pungas 2017; Pungas et al. 2022, p. 137; Sovová and Krylová 2019).

With regard to FSP as an agricultural practice, Western scholarship (but also Estonia’s own political elite) fails to recognize the value of FSP as a result of postcolonial prejudices (Pungas et al. 2022). Yet, at the same time, Estonia promotes the same agricultural bioeconomy policies as the EU, which will—in a neo-colonial way—exacerbate already existing profound global injustices by prioritizing the global expansion of its agricultural produce. The Estonian agri-bioeconomy paper’s only vague reference to the development aid is for instance not motivated by the recognition of global colonial injustices (such as ecological debt), but by the calculated goal of creating “a positive image [..] as a prerequisite for exporting products and services to new markets” (Agri 2021, p. 9). There is no indication of Estonia’s extremely high carbon footprint (Estonia is the most carbon intensive economy in the OECD due to oil shale, OECD 2017) or high consumption levels. Instead, prosperity and competitive advantages on global agricultural markets seem to be the sole focus, to which “climate change might even contribute to” due to better weather conditions (Agri 2021, p. 9). These narratives not only ignore the present global challenges and injustices, but instead promote their further exacerbation.

### Capitalist nature relations—bioeconomy models deepening the human–nature dichotomy?

The second sphere “Capitalist nature relations” illustrates that nature^6^ is regarded in capitalism as a mere matter, a ‘passive reservoir’ full of raw material, free for unrestricted appropriation. In a comparable manner, the EU bioeconomy strategy reproduces “neoliberal imaginations of the economization and commercialization of nature that reinforce technology- and growth-centered bioeconomy visions” (Vogelpohl and Töller 2021, p. 145; Hausknost et al. 2017). Nature’s function then is to serve humans and their economies, either by providing the material basis (e.g., biomass), delivering natural assets, or offering aesthetic joy and well-being in untouched nature ‘reserves’ in which the “exhausted and alienated wage-workers could regain their humanity” (Mies et al. 1988, p. 5; Schmidlehner 2021).

The currently promoted bioeconomy models deepen the human–nature dichotomy due to capitalist (societal) nature relations (Görg 2004; Burandt 2018), which manifest themselves in an almost unquestioned conviction of the endless availability of and right to access natural resources for humanity’s sake. As such, the root causes of ecological crises, such as the “imperial mode of living” (Brand and Wissen 2021) and the overconsumption of nature (e.g., deforestation for raw biomass, intensive livestock farming along with carnivore diets) remain unaddressed. The only difference to previous economic models seems to be the focus on ‘renewable’ biological resources to replace fossil fuels—however, they are still regarded as infinite (Mills 2015, p. 23). Furthermore, ecological processes such as sources and sinks as well as the regeneration of nature seem to be ‘taken for granted’; they are perceived as ‘natural’ and self-evident, and thus remain ‘invisible’ (Mies 2007; Dengler and Strunk 2018). This (in)visibility of one in comparison with the other can be exemplified with humus-rich soil, which constitutes the material basis for the agricultural sector: the ‘natural’, ecological process of soil generation and humus formation (e.g., via composting practices in FSP) has no monetary price (and is not compensated by monetary means), even though the soil as a monetized ‘end product’ has also become an increasingly valuable commodity in the world market (see, for example, Plank 2013). Another new mechanism of nature appropriation can be observed in the commodification of nature via ‘ecosystem services’. Quantified and consequently inter-changeable ‘services’ make it possible to extract commercial value from nature by simply redefining these services as internationally tradable speculative commodities—“without anything being physically extracted or produced, ‘financial assets’ are created from the land in the form of certificates” (Schmidlehner 2021; Farrell 2014).

The destructive antagonistic relationship of humans with the more-than-human world, made increasingly evident by the concurrent and exacerbating ecological crises, is part of the capitalist economic system and is deeply rooted in the Western notion of nature (De Groot 1992; Descola and Sahlins 2013). This human/culture–nature dichotomy, in which nature as the fundamental alterity (the ‘other’) constitutes the Western Man (the ‘I’) (Schmidlehner 2021), enables and legitimizes human dominance over nature. In the name of enlightenment and development, ‘nature’ has been demystified to a mere object (Merchant 1980), as opposed to a subject—possibly even as a legal personhood with rights of its own—as in the case of indigenous and rural communities in various peripheral countries (e.g., *buen vivir* and ‘rights of nature’ in Ecuador and Bolivia, sacred rivers in Colombia and India, or birch tree hugging and the so-called ‘soil religion’ in Russia, see Berdyaev 1907; Jeffreys 2021; La Follette and Maser 2020; Ramírez 2020). Bioeconomy visions based on diverging relationship models with nature (e.g., partnership, care, and mutual respect instead of utilitarian mastery and dominance of nature, Pungas 2022) are systematically devalued (Longhurst and Chilvers 2019; Priefer et al. 2017; Levidow et al. 2012), and instead the high-tech oriented knowledge-based bioeconomy along with diverse ‘novel’ technologies such as biotechnology (e.g., bio-based chemicals, pharmaceuticals and plastic, along with certified seeds) (EU 2018, p. 32, 46; Agri 2021, p. 40) seems to be the major promise of EU and Estonian bioeconomy papers alike (EU 2018, p. 5, 41ff; Tiits and Karo 2021, p. 6, 8; Tiits et al. 2021, pp. 33–35). In these areas, unique opportunities for economic growth, new export markets and ‘value’ creation are supposedly awaiting (ADDVAL-BIOEC 2021; Tiits and Karo 2021, p. 6, 8, 10; Tiits et al. 2021, pp. 33–35; Goven and Pavone 2015).

As such, it is not surprising that inefficiency—rather than high economic output—is seen as the main cause for most ecological problems to which natural science and technological innovation, along with circularity and the persistent and problematic belief in decoupling, are to provide solutions (Birch et al. 2010, p. 2898; Giampietro 2019; Bugge et al. 2016; Levidow et al. 2019; Levidow 2011, p. 7, 9). This results in various small-scale, low-tech economic activities based on alternative human–nature relationship models being disregarded or overlooked. Due to their apparent ‘inefficiency’, their crowding-out is legitimized.

### Capitalist nature relations: “FSP is an inefficient use of natural resources”

If we explore the invisibility of certain bioeconomy practices such as FSP in CEE through the ‘capitalist nature relations’ sphere, the line between visible and invisible bioeconomy runs between the monetized/quantifiable nature (nature as a resource or commodified ecosystem services) (EU 2018, p. 4) and the realm of ecological processes that take place beyond the market. This boundary between the economic and non-economic in the bioeconomy can be identified in its continuous “attempt to incorporate and capitalize the ecological” (Goven and Pavone 2015, p. 307).

As such, the FSP practice is clearly a non-monetized agricultural practice, perceived as an ‘inefficient’ subsidiary in contrast to alleged ‘progressive’ and ‘efficient’ large-scale farm enterprises (Mamonova 2018). As the large variety of their socio-ecological benefits are not counted or indeed non-quantifiable, enhanced biodiversity, pesticide-free garden produce (and its sharing among family and friends) and nutrition, composting, green manure and other practices go unnoticed. Furthermore, as various scholars have demonstrated, the ecological benefits and the value of the FSP practice might not even be acknowledged by the gardeners themselves, either because they are regarded as ‘common sense’ or because gardeners have a disparaging attitude toward their own farming practices (Visser et al. 2015, p. 523).

In comparison with industrial, profit-maximizing farming methods, FSP practitioners enhance soil quality, protect biodiversity, produce considerably less GHG, and use barely any mineral fertilizers or chemical pesticides (Burandt und Mölders 2017, p. 962; Vávra et al. 2018; Pungas 2019). In contrast to the EU and Estonian bioeconomy papers, that promote technological innovation and increased efficiency (meaning more sophisticated ‘mastery’ over natural resources), FSP gardeners and subsistence farmers demonstrate different relationship models toward nature (such as stewardship and partnership) (Pungas 2022), and their practice is mostly motivated by ethics of care for nature (instead of exhausting its resources for maximum profit) and food (Pungas 2020; Mincyte et al. 2020; Sovová et al. 2021).

Hausknost et al. (2017) have differentiated between contrasting bioeconomy visions diverging between (1) sufficiency and growth and (2) agroecology and biotechnology orientations. They contend that EU and OECD bioeconomy strategy papers have a clear growth and biotechnology bias (called “sustainable capital”), whereas the counter pole (called “eco-retreat”) is characterized by principles of sufficiency and agroecology. FSP practice serves as a perfect example for the latter. However, as it does not comply with the dominant bioeconomy paradigm, it also remains unacknowledged in the Estonian bioeconomy papers.

Furthermore, even ‘visible’ corporate organic agriculture, which would contribute to the economic growth of the bioeconomy sector, is barely mentioned—and if so, only as a necessary future research and investment area (EU 2018, p. 48; Agri 2021, p. 56). Despite deteriorating eutrophication levels in the Baltic Sea (mostly due to the over-use of nitrogen and phosphor-based fertilizers) (Tóth et al. 2014), local organic agriculture receives no further subsidies nor other competitive advantages, while the negative impacts of the conventional agri-food system (fertilizers, pesticides, GHG) are only marginally—if at all—addressed (Agri 2021, p. 8). Instead, the Estonian agri-bioeconomy paper foresees, among others, the increase of livestock farming and biomass production, although scientists have highlighted the incompatibility of such intensified agriculture with environmental sustainability goals, and demand, for instance, a ¾ reduction of livestock farming (Monaghan 2021; Ekardt et al. 2021, p. 24).

### Patriarchy—bioeconomy models maintaining the separation between monetized and maintenance economies?

The third sphere of devaluation and appropriation in the current economic model is patriarchy. As a more comprehensive analysis on the variety of manifestations and aspects of patriarchy exceeds the scope of this article, I conceive it broadly as a power relation over women (including their bodies and labor) that is justified by inherent differences between the two genders. Feminist scholars and activists have problematized women’s subordination, suppression, or exclusion throughout history (examples include the Suffragette’s campaigns for the right of women to vote and own property in the early twentieth century, Simone de Beauvoir’s groundbreaking ‘The Second Sex’ from 1949 (de Beauvoir 1949, 1972), which gave rise to the struggle for women’s reproductive rights and intersectional feminism). Despite the subtle and complex spectrum of ongoing manifestations of patriarchy in nearly all societies, in this article I focus on the structural separation between wage and household labor as a common characteristic of all patriarchal societies, that results in an exclusive focus on wage labor in their economics^7^ and makes all other forms of labor practically invisible (Mies et al. 1988; Himmelweit 1995; Haidinger and Knittler 2014; Haug 2009).

Women are considered as the “last colony” (1988) by the authors of the Bielefeld subsistence approach, since the mostly female household labor is appropriated by capitalist accumulation yet not considered as an integral part of macroeconomic analyses (Mies et al. 1988). According to them, women have been ‘naturalized’ or placed within the ‘realm of nature’, the same way as colonies and living nature have been (Mies et al. 1988, pp. 4–5): “Women [and subjugated peoples] are treated as if they did not belong to society proper, as constituted from (male) wage-workers and capitalists. Instead, they are treated as if they were means of production or ‘natural resources’ such as water, air and land” (Mies et al. 1988, p. 5). This process of systematic subordination of the reproductive or maintenance sphere (or care work) (Burandt and Mölders 2017) in comparison to the production (or monetized) sphere is, according to Marxian feminist political economy, the necessary precondition for the global capitalist patriarchal model of accumulation (Biesecker and Hofmeister 2010; “Wages Against Housework” by Federici 1975, 2004; Saave 2022). These arguments have, to some extent, gained traction due to an exacerbating ‘care crisis’, especially during the COVID-19 pandemic, with regard to the so-called ‘essential workers’ (Koebe et al. 2020; Dowling 2021a, b; Care.Macht.Mehr 2013).

According to the Bielefeld subsistence approach, the ‘flexibilization’ of labor (the so-called “housewifized labour”, also among men) will increase also in the ‘Global North’, because formal wage labor will either be too expensive or not productive enough (Mies et al. 1988: 10). “The proletarian disappears” (Mies et al. 1988, p. 10, 170ff.) and will be substituted by informal^8^ labor, that, similar to domestic labor, is neither protected by trade unions nor labor laws, “available at any time, for any price, […] not recognised as ‘labor’ but as an ‘activity’, [..] isolated and unorganized” (Mies et al. 1988, p. 10, 175). Such ever-increasing cheap, non-unionized, and privately managed labor can be observed in the gig-economy, in agriculture (e.g., Europe's transnational agribusinesses depending on the low-wage East-European seasonal workers in the fresh vegetable and meat sectors, see Cosma et al. 2020), but especially in care sectors (“global care chains”, Yeates 2004). The global COVID-19 pandemic, along with home-offices and home-schooling, intensifies the “old/new strategy of homeworking”, that Bielefeld scholars warned about as early as the 1980s (Mies et al. 1988, p. 10). They argue that this tendency has not only economic but also political motives and consequences, as it will break the power of organized wage labor (trade unions) in an increasingly atomized home–office–society.

The same above-mentioned characteristics that enable the devaluation and appropriation of housework and other reproductive activities (child and elderly care) also apply for various forms of (non-wage) work and subsistence (including subsistence farming). Despite the fact that the majority of the global population is engaged in different forms of subsistence, the predominant development discourse regards subsistence as an inefficient and ‘backward’ activity (in contrast to formal wage labor) (Bennholdt‐Thomsen 1982). At the same time, however, Mies et al. (1988) argue that, in a manner comparable to (female) housework appropriation, subsistence resources and work in the Global South were discovered by international finance and ‘development’ agencies as an ‘untapped potential’ to be exhausted in a ‘productive’ way for the accumulation process (Mies et al. 1988, p. 7). Contrary to the claimed promise of poverty alleviation among rural subsistence producers, the authors confirm the previous findings of the central critics of capitalist agriculture, namely, Marx (1978) and Chayanov (1966), who argued that the further integration of subsistence life and economy into the market economy exacerbates the living standards of the subsistence producers, which according to Bielefeld subsistence approach is “the inevitable effect of capitalist modernization” (Mies et al. 1988, p. 7, 40ff.).

The bioeconomy papers reproduce the same structure of separation between the productive and reproductive sphere that has been criticized by feminist ecological economists. Different forms of ‘informal’ labor are neither mentioned nor acknowledged as the foundation of wage labor (Gibson-Graham 1997; Mies 1998). Although food and agriculture are considered the largest sectors of the bioeconomy (in the EU 71% of all value-added bioeconomy and 76% of employment, FAO 2022; EU 2018, p. 29), it is ignored that globally (but also Europe-wide, EC 2020) most of the work in these ‘sectors’ happens outside of wage-labor relations: family farms constitute over 98% of all global farms (Graeub et al. 2016) and housework around food/nutrition (purchasing food, preparing meals, feeding children/elderly) constitutes the lion’s share of food-related reproductive work. This supposed ‘naturalness’ and normality of social relations that makes one type of labor visible while invisibilizing the other demonstrates the persisting patriarchal power relations within current bioeconomy models and has to be regarded as such (Mies et al. 1988).

### Patriarchy: “FSP is not a (valuable) labor”

Within the sphere of patriarchal power relations, all non-monetary forms of labor are systematically overseen, devalued, marginalized, and downgraded to mere ‘activities’. This results in the neglect of all forms of labor and practices that happen outside the market, despite the latter being globally far more widespread. Despite the prevalence of FSP in the CEE region (but also globally), its multiple socio-economic and ecological benefits, as well as its significance for the life quality and well-being of the practitioners, it does not exist ‘on paper’. Such a narrow lens, that only regards market-based labor, thus omits a broad spectrum of “diverse economies” (Gibson-Graham 2008; Cima and Sovová 2022) and ‘informal’ practices that thrive outside the sphere of the state and the formal market (Morris and Polese 2015).

Both FSP and (semi-)subsistence farming are devalued by a similar patriarchal logic: the labor and care given to plants, soil, family and community members are ‘feminized’ and subsequently devalued as ‘natural’ activities (as opposed to ‘unnatural’ wage labor) which neither need a financial compensation, legal protection nor deserve symbolic acknowledgement (Martin 2019, p. 104). Such ‘naturalized’ labor of care, which is—supposedly—performed merely for pleasure, ‘instinctively’ or as a recreational activity, enables further devaluation of this physically and psychologically challenging and skilled work (Burandt und Mölders 2017, p. 962). Although the ethics of care indeed prevail among FSP practitioners as an intuitive common sense, it should not make it less valuable. Still, what counts as part of the bioeconomy in the agricultural and food sectors is exclusively wage labor—subsistence, FSP, care, reproductive and domestic labor are neither mentioned nor acknowledged in the respective bioeconomy papers.^9^ Therefore, bioeconomy papers reproduce the very same power relations and invisibility of certain spheres by devaluing and disregarding one (labor) over another.

Furthermore, through the devaluation process, such practices and labor can potentially be appropriated and co-opted by the neoliberal economic system. This can happen with all types of urban agriculture (FSP being just one of them) and can take many forms, as various scholars have shown (Pungas et al. 2022; McClintock 2014). Through “green gentrification” (Gould and Lewis 2016), “community capitalism” (Van Dyk 2018), and the “sharing economy” (Martin 2016), the current economic model has found creative ways of co-opting devalued practices, civic engagement, and neighbourhood initiatives as services and labor provided ‘for free’ by activists, community members, and ‘active citizens’ alike.^10^ FSP can thus unintentionally become a buffer mechanism to compensate for the shortcomings of the neoliberal governance, and thus end up indirectly maintaining this system (Pungas et al. 2022).

In addition, there is an indispensable ‘grey zone’ between wage and non-wage labor. In this informal sector, labor is only compensated minimally and neither enjoys the protection of labor laws (as in the case for the gig-economy) nor societal appreciation. With regard to the bioeconomy, such informal labor prevails in the agricultural sector (e.g., migrant/seasonal workers for asparagus in Germany, see Cosma et al. 2020, or in the tomato farms around Almeria, Gertel and Sippel 2014), in the so-called “global care chains” (Yeates 2004), or in the construction sector (e.g., Ukrainian construction workers in Western Europe, Vershinina et al. 2018; Boatcă 2013).

Another paradigm that characterizes the patriarchy sphere is the public–private divide, which has consequently resulted in the shifting of ‘public’/state responsibility to the ‘private’/personal (or non-state) sphere of single individuals (predominantly regarded as consumers) via various market mechanisms (Nicholson 1986; Boyd 1997; Jennings 1993; Dengler and Lang 2021). In the Estonian agri-bioeconomy paper, citizens are reduced to mere consumers (or alternatively, producing entrepreneurs) who only require adequate environmental information to make conscious choices at the market. Correspondingly, the produced food is valued merely with regard to market requirements (Agri 2021, p. 6, 16). This not only ignores extensive social science research on the behavioural gap theory, but also disregards FSP practitioners as active, sovereign, and self-reliant producers (as opposed to mere consumers) which do not depend much on the market.

## Conclusions

With the help of the framework based on the Bielefeld subsistence approach, I have not only demonstrated the ‘blind spots’ of the EU and Estonian bioeconomy policy papers, but also elucidated how the three-dimensional devaluation and appropriation of these invisible spheres can explain why such bioeconomy practices as FSP remain invisible in all relevant policy papers. First, it is through postcolonial logic that FSP practices are crowded out and respective skills and knowledge are neglected as outdated and backward. Second, in capitalist nature relations eco-centric, agro-ecological and sufficiency-oriented practices such as FSP are disregarded as inefficient, small-scale, and unprofitable despite their socio-ecological benefits. Third, according to patriarchal logic, FSP as a non-monetized or ‘informal’ labor in the ‘private’ sphere is regarded as a mere (valueless) activity. As such, the current bioeconomy models not only mobilize for the bioeconomy as a political project of continued capital accumulation by generating consensus with their promissory discourse, but they also suppress and sideline efforts that actually address the underlying root causes of the problems the bioeconomy claims to solve. Or as Salleh (2010) puts it: “Of course, a true bio-economy will only be found at the peripheries of capitalism, but that is not appreciated where the faith in technology rules” (Salleh 2010, p. 209).

If the bioeconomy truly wants to have sustainability “at its heart” (EU 2018, p. 4), the only suitable model for it is that of ‘strong sustainability’, which actively, and in all policies, recognizes the ecological foundation of all social and economic activities, and consequently recognizes the physical and material boundaries of the latter. Genuine consideration of both, social and ecological boundaries in bioeconomy policies would, therefore, do justice to the foundational idea of the concept of ‘bio-economics’ by Georgescu-Roegen. For this endeavour, it is crucial to engage with the underlying capitalist, (neo)colonial, and patriarchal power relations of current bioeconomy models that devalue and appropriate the above mentioned three respective spheres of the bioeconomy for the sake of further capital accumulation and expansion.

Furthermore, if the imperative is to “improve and innovate the way we produce and consume food” (EU 2018, p. 4), why not acknowledge and nurture already existing diverse practices that do so in sustainable and convivial ways? The European bioeconomy strategy states: “Food and farming systems are a fundamental part of the bioeconomy, but they urgently need to be transformed to become more sustainable, nutrition-sensitive, resilient and inclusive in view of a growing world population, climate change and other environmental challenges, including water scarcity and loss of biodiversity and of productive land” (EU 2018, p. 26). Therefore, suggestions for a genuinely transformational bioeconomy, inspired also by the practice of FSP, include: first, to deconstruct the current bioeconomy models as just another postcolonial development discourse and instead embrace the plurality of decolonial ‘alternatives to development’; second, to overcome the deepening human–nature dichotomy in current bioeconomy models and instead cultivate mutually nourishing, partnership-like relation(ship)s with nature; and third, to foster ethics of care in order to overcome the structure of separation between monetized and maintenance economies. Instead of remaining an object of three-dimensional devaluation and appropriation through dominant bioeconomy models, FSP could instead become an inspirational example for designing a socially just and ecologically sane bioeconomy.

## Data Availability

The authors confirm that the data supporting the findings of this study are available within the article.

## Footnotes

1 See Collard and Depsey (2020) for various applications of the iceberg metaphor.

2 “Quiet sustainability” as a concept summarizes, according to its authors, “widespread practices that result in beneficial environmental or social outcomes and that do not relate directly or indirectly to market transactions, but are not represented by their practitioners as relating directly to environmental or sustainability goals” (Smith and Jehlička 2013). Building on this concept, Visser et al. (2015) coined the term “Quiet Food Sovereignty “ when exploring the ‘thriving’ of traditional small-scale farming practices in post-socialist Russia through the lens of food sovereignty. The authors conclude that smallholders share the visions and ideas of the global food sovereignty movement, although the political dimension and discourse on rights and entitlements are rather implicit among smallholders (Visser et al. 2015) in comparison to the Nyéléni (2007) declaration.

3 Instead of “Capitalism”, I have named the respective sphere of the colonialism-capitalism-patriarchy nexus “capitalist nature relations”. I join ecofeminist and ecosocialist feminist scholars who argue that it is only within capitalist nature relations that nature becomes the third devalued and appropriated foundation of the economic system (Mies et al. 1988: 96ff; Salleh 1997; Mellor 1996), whereas colonialism and patriarchy are already by definition mechanisms of oppression and domination. Furthermore, with regard to capitalist (societal) nature relations, nature is neither regarded in a comparable manner in all economic activities, nor by all persons or communities (see Foster et al. 2011: 251ff; Foster 2022; Brand and Wissen 2018 and Görg 2004).

4 It is acknowledged here that the emancipation of peasant(s or) women or the working class is also seen as part of the benefits brought or advocated by the very same ‘modernity’.

5 It is acknowledged here that there are radical inequalities within the populations of the countries of the ‘Global North’, the ‘Global South’ (and ‘Global East’) where exploitative class relations vary significantly. However, in this article, I join the scholars who argue that global material flows have been unequal towards, and “draining” the ‘Global South’, as shown by Hickel et al. 2022.

6 While acknowledging that nature as a concept is socially produced and manifests itself in the societal nature relations (Görg 2004), the biophysical materiality of nature remains the basis for means of production.

7 Herewith I acknowledge that unpaid “women's” work is neither done by all women nor only by women.

8 According to von Werlof, the respective ‘informal sector’ also bares names such as “part-time work, contract work, seasonal and migrant work, illegal work, ‘borrowed’ work, as well as unpaid work like the so-called ‘work for one’s own’ (v. Weizsäcker), ‘shadow work’ (Illich), subsistence work and, mostly ‘forgotten’, housework; in short, generally speaking (wage) labour that is not ‘free’.” (Chapter “Proletarian is Dead: Long Live the Housewife” by Claudia von Werlof in Mies et al. 1988: 171).

9 To my best knowledge, none of the current bioeconomy policy papers have addressed this issue. However, there are various publications on comparable topics, such as D’Alisa and Cattaneo 2013 and Saave et al. 2022.

10 Non-commodified agriculture/food related examples include the wide variety of community gardens, community kitchens, and initiatives/platforms against food-waste such as Foodsharing, whereas ‘Too good to go’, as a monetized mobile application with the same goal of avoiding food waste would serve as a counter example.
